# Where boys don’t dance, but women still thrive: using a development approach as a means of reconciling the right to health with the legitimization of cultural practices

**DOI:** 10.1186/s12914-020-00230-2

**Published:** 2020-06-15

**Authors:** Melisa Demir, Bilkis Vissandjée, Danielle Jacobson, Gillian Einstein

**Affiliations:** 1 McGill University Faculty of Law, New Chancellor Day Hall, 3644 Peel street, Montréal, QC H3A 1W9 Canada; 2 School of Nursing, Université de Montréal, 2375 Côte Sainte Catherine, Montréal, QC H3T 1A8 Canada; 3 SHERPA Community-based Research Centre, CIUSSS West-Central Montreal, 7085, rue Hutchison, Montréal, QC H3N 1Y9 Canada; 4 Dalla Lana School of Public Health, University of Toronto, Toronto, ON M5S 3G3 Canada; 5 Department of Psychology, University of Toronto, 100 George St, Toronto, ON M5S 3G3 Canada; 6 Department of Gender Studies, Linköpings Universitet, Linköping, SE-581 Sweden

**Keywords:** Gender inequity, Cultural norms and practices, Women’s health, Human rights discourse, Female genital cutting/excision

## Abstract

Human rights language has become a common method of internationally denouncing violent, discriminatory or otherwise harmful practices, notably by framing them as reprehensible violations of those fundamental rights we obtain by virtue of being human. While often effective, such women’s rights discourse becomes delicate when used to challenge practices, which are of important cultural significance to the communities in which they are practiced. This paper analyses human rights language to challenge the gender disparity in access to health care and in overall health outcomes in certain countries where such disparities are influenced by important cultural values and practices. This paper will provide selected examples of *machismo* and *marianismo* discourses in certain Latin American countries on the one hand and of female genital cutting/excision (FGC/E) in practicing countries, both of which exposed to women’s rights language, notably for causing violations of women’s right to health. In essence, a reflective exercise is provided here with the argument that framing such discourses and practices as women’s rights violations. Calling for their abandonment have shown that it may not only be ineffective nor at times appropriate, it also risks delegitimizing associated discourses, norms and practices thereby enhancing criticisms of the women’s rights movement rather than adopting its principles. A sensitive community-based collaborative approach aimed at understanding and building cultural discourses to one, which promotes women’s capabilities and health, is proposed as a more effective means at bridging cultural and gender gaps.

## Background

The sun is setting outside a bedroom window in Lima, Peru, when the quiet sound of a child’s footsteps rings through the doorway. A little boy slams his two tiny hands onto the table in front of him. His mother watches as he dances along to his favourite song, his feet perfectly placed in first position, his posture poised enough to mirror that of a budding ballerina.

“He would be such a great dancer,” she says with a glimmer of pride in her eyes. “But his father would never allow it. Boys don’t do that here.”

Indeed, Peruvian culture - similar to that of many Latin American nations - remains imbued with pervasive gender roles, which delineate the socially accepted boundaries within which within which boys, men, girls and women are expected to act [1]. They shape Peruvian identity and instill a power hierarchy in relationships, particularly in communities with lower socioeconomic status [2], thereby creating marked by gender inequities – where boys and men don’t dance, and girls and women stay home [3].

Literature has consistently shown the harmful impacts of this gender-based power dynamic on on girls and women within Latin American societies, particularly concerning their health [1–3]. One example which was demonstrated over and over again is that norms often act as barriers for human immunodeficiency virus infection and acquired immune deficiency syndrome (HIV/AIDS) prevention in Latin American women [1, 3]. This is one example of how gendered practices in some countries^1^ can be detrimental to girls’ and women’s health^2^. Such gender norms create an environment where girls and women in certain Latin American countries, in order to comply with expected gender roles, often fail to seek necessary medical attention, placing the health and wellness of the rest of their families above their own [1]. Such gender norms leading to detrimental health outcomes for women exist globally [4]. Similarly, some cultural practices in certain countries limits womens’ autonomy and ability to do so on their own volition [5].

Selected gender practices perpetuate health inequities in societies within which harmful practices operate, acting as a barrier to positive health outcomes, particularly women’s empowerment in the area of reproductive health [1, 6–8]. In this regard, our argument builds on the importance of a proper understanding of the contribution of cultural practices in its perpetuation [9]. The discourse around legitimacy of practices, labelling them as women’s rights abuses risks contributing to an undue perception of women’s rights discourse as a top-down, ‘Western ideals’, imperialistic narrative [10–13]. Cultural and gender sensitive frameworks will reduce the incompatibility of policies and allow them to be more effective [6, 7].

We consider the examples of *marianismo* and *machismo* cultures in selected Latin American countries and of female genital cutting/excision (FGC/E) in practicing countries^3^ to explore the contribution of cultural discourses in the perpetuation of gender-based health inequities. In essence, a reflective exercise is encouraged with a core argument that framing such discourses and practices as women’s rights violations needs careful attention. Calling for outright abandonment may not only be ineffective nor at times be appropriate. It risks delegitimizing associated discourses, norms and practices thereby enhancing criticisms of women’s rights movement rather than rather than understanding and adopting its principles. A sensitive community-based collaborative approach aimed at understanding and building cultural discourses to one, which promotes women’s capabilities and health, is proposed as a more effective mean in an attempt to bridging cultural and gender gaps. The focus on what women are “actually able to do and be” ([14] p.33), can more effectively advocate for women’s health by creating a gender-sensitive environment - a ground where boys and men may not dance, but girls and women can still thrive.

## Cultural discourses, norms and practices as drivers of gender inequality in health conditions

According to the United Nations, half of the women in selected countries do not have access to the health care they need and female life expectancy at birth is still considerably low [8, 15]. In this regard, our arguments focus on the importance to focus on cultural discourses, norms and practices that adversely affect women’s health and prevent them from seeking the medical attention they need [2].

Our analysis addresses how pervasive gender norms are in selected countries in Latin America contribute to the increased risk in women contracting HIV/AIDS. Similarly, we propose a reflective practice on how cultural meanings of FGC/E in practicing countries contribute to its perpetuation. The central point is that culture, traditions and discourses often lie at the root of the inequities in health conditions.

### The consequences of *Machismo* and *Marianismo* cultures in Latin American countries on the prominence of HIV/AIDS in women

The cultures of *machismo* and *marianismo* embed pervasive gender roles into the social discourse of many Latin American countries^4^ [3]. Together, they create a gendered belief system which promotes the dominance of masculine ideals within a given Latino society [16]. This cultural narrative centred around principles of male dominance and female passivity, is linked to an increase in the prevalence of HIV/AIDS in Latin American countries, mainly due to the values it promotes and the aspirations it outlines for men and women early on in their lives [17].

*Machismo* culture refers to the expectation that men are meant to be strong, active, and independent actors in the family [1]. It additionally promotes the idea that men must have multiple sexual partners, both before and after marriage [3]. Conversely, *marianismo* refers to the woman’s expected role in society as one that embodies the values of modesty, chastity, and the willingness to serve men [16]. It places a strong emphasis on women’s submission to men both within the family and with regards to sexuality. Indeed, women in *marianismo* cultures are expected to forego their decision-making skills and autonomy in the family life and to remain “sexually naïve,” ignorant of healthy sexual practices such as the importance of contraceptives and condom use [1]. In essence, *machismo* and *marianismo* cultures work together to support an open hierarchical gender structure within society where men are granted freedom, autonomy and decision-making power, particularly in the family and in sexual relationships, while women should remain passive, submissive and ignorant of safe sexual practices [1].

This hierarchical belief system created through the intersection of *marianismo* and *machismo* value sets is strongly ingrained in Latin American culture, to the extent that the ideals and expectations it promotes become constructive of the Latino and Latina identity [17]. In selected societies in Peru, for instance, male and female identities are typically classified as either “*macho*/*mariana*,” or as “*maricon/puta”*^5^ [1]*.* The former classification refers to men and women who conform to their socially accepted roles and, therefore, benefit from widespread social acceptance [1]. In contrast, men and women who deviate from their socially expected gender roles – for instance, men and women who are knowledgeable about sex and particularly sexually experienced - are pejoratively labeled as *maricones* and *putas*. Those whose identities fall into these latter categories often find themselves at the center of widespread humiliation and social rejection [1].

As a result, Peruvian men and women strive to meet these gender expectations in order to benefit from socially sanctioned identities [1]. In addition, such societal attitudes act as an obstacle in HIV/AIDS prevention, as it promotes unhealthy sexual behaviour for men and, more importantly, prevents women from negotiating healthier practices. Men in *machismo* cultures may engage engage in extramarital affairs as a means of proving their virility, and thus, to remain in good social standing [1]. These affairs frequently occur without condom use, thereby increasing the risk of HIV/AIDS transmission [1]. Moreover, the expectation that *mariana* women remain submissive to men gives them little say in the sexual relationship, and would likely render their requests for condom use futile [1, 17]. HIV/AIDS in women in these cultures is similarly heavily stigmatized. As a result, the fear of being viewed as overly sexually experienced or knowledgeable - and thus being classified as a *puta* and bearing social rejection and humiliation - often prevents women from seeking the testing, treatment and medical care they need to live with a potentially deadly condition [1]. Lastly, a *machismo/marianismo* centered society promotes the idea that women must place their needs second to the interests of their husband and children [1]. Therefore, their seeking necessary healthcare is rare [3].

The effects of a tarnished reputation due to a disclosure of one’s HIV-positive status within these societies are particularly difficult on women, who often cannot afford to be financially rejected by their families [1]. As a result, upholding their socially sanctioned identities is crucial. As indicated above, doing so can be equally harmful, as it requires women to engage in and accept sexual practices which increase their risk of contracting HIV/AIDS, and continue to obstruct to their access to necessary health care.

A gendered discourse similar to that arising from the cultural values and traditions in Latin America equally exists in selected countries, nearly all of which are classified by the World Bank as low-middle income countries [18]. Indeed, social expectations akin to those of *machismo* and *marianismo* cultures – namely, that women remain virgins until marriage [6] and live as housewives [9] - are still prominent in a number of countries^6^. Such expectations associated with socially sanctioned identities for boys, girls, men and women, much like *machismo* and *marianismo* values set a standard for what is considered “proper sexual behaviour” [19, 20].

### The consequences of gendered socio-cultural expectations of female genital cutting/excision (FGC/E) on girls and women in selected communities of origin and in migrant host societies

While questioned by a diversity of women’s organizations, the most common definition, provided by the WHO, refers to FGC/E as procedures that intentionally alter or cause injury to the female genital organs for non-medical reasons [18, 19]. This happens on girls between the ages of five and eight, but can be performed as early as the first week of life and as late as the time of marriage [19].

The rationales behind this continued practice vary from community to community. It is viewed, by some, as a means of preserving a woman’s pre-marital virginity, central to their identity and acceptance both within their families and within society. Some others view this practice as an essential rite of passage, which allows young girls to prepare for their transition into womanhood [9]. The pain inflicted on girls during the process is seen to be a necessary preparation for the daily hardships of adult life, as well as preparation for the pain of childbirth [9]. While FGC/E practices related arguments vary, rationales invariably remain imbued in patriarchal social expectations and perpetuated by long-standing cultural values, in and itself a manifestation of gender inequities [21, 22]. Social exclusion and a risk of physical violence are documented among those who ‘should have and were not’ exposed to this practice. In addition, reports of uncircumcised wives being cast out of their communities, of women being shunned by their families for disgracing the family honour by refusing to undergo the procedure, or of young girls seeking the procedure in order to better conform with their classmates and fit in to their social surroundings are well documented [9, 19]. The pervasiveness of FGC/E practices is often heightened by a strong social pressure to conform to expected identities, on the one hand, and by women and girls’ poignant desire to belong to their community, on the other [19]. While there are no documented health benefits, the severity of health risks is associated to the type of the procedure performed [19–22], usually by elder members of the community who are considered “guardians of the tradition” perceived most fit to carry out the operation, a ceremonious rite of passage [9]. Most common consequences of the practice of FGC/E on girls and women include serious psychological disorders [19] as well as physical pain during menstruation, sexual intercourse, higher likelihood of contracting bladder and urinary tract infections and an increased risk of encountering complications in childbirth [20].

Given that FGC/E procedures are heavily embedded in cultural narratives, it has left policymakers on both national and international levels in a form of catch-22, in which strategies need to address the cultural importance given to FGC/E and its social, legal and health consequences [9]. Deeply rooted cultural discourses, norms and practices are pervasive and may not respond to policies and legislation. Finding the appropriate balance between recognizing the cultural and traditional significance of these actions that support community norms and protecting girls’ and women’s health is fundamental in order for policies to be truly effective. Such balance is not easy to strike. It requires a respectful and gender-sensitive discourse as a foundation.

## Women’s rights discourse: a potentially ineffective means of reconciling means of reconciling girls and women’s health discourse with prevalent cultural norms

Human rights are the rights one is entitled to for simply being born [23]. They are, in principle, universal and inalienable to all by virtue of being human. A human rights-based discourse promotes or condemns behaviour based on its alignment with selected fundamental entitlements – such as the right to equality before the law, the right to be free from torture and degrading treatment, or the right to the highest attainable level of health - acquired simply based on our status as humans [24]. This narrative has been dominant within the international community since the mid-twentieth century. Such statements become particularly relevant when considering how policies rooted in human rights discourse interact and intersect with traditional practices ingrained in a society’s cultural values [11]. Human rights narratives are largely influenced by Western values with a risk of misunderstanding issues when discourses lack sensitive gendered analyses. While rights-based solutions were conceived with good intentions, tackling cultural discourses and values ingrained within a society, rights-based solutions may not work as well as they intend to.

### The right to health and its rise to international prominence

The idea of human rights as we conceive it today rests within an international framework, made up of treaties and declarations setting out standards that countries are expected to uphold. The idea of promoting respect for fundamental rights in order to ensure a better quality of life has existed throughout history, long before it rose to prominence on an international scale^7^. The recent conception of human rights discourse as one resting in international agreements and cooperation stems from the post-World War II era, marked by great optimism and a widespread desire for international peace and stability [23]. During this era, the human rights language 'went global' [25] p. 1420). This happened as the United Nations was created in 1945 and the Universal Declaration of Human Rights was being drafted in 1948 [23, 25]. One of the main goals of this latter initiative was for countries to work together to create a universal standard of morality in an effort to reconcile with diverse with their diverse cultural and political backgrounds. While Western countries had greater influence over the language of the Declaration, international cooperation and a desire to achieve justice and peace were nonetheless central to its adoption [25].

Among these protected entitlements is the right to the enjoyment of the highest attainable standard of physical and mental health, more commonly known as simply ‘the right to health,’ which finds itself at the center of the debates surrounding the cultural practices highlighted in this paper. It encompasses a number of freedoms and entitlements - such as the right to be free from cruel, inhuman or degrading treatment and the right to equal and timely access to basic health services - outlining the importance of health in ensuring a dignified life [26]. It does not, however, impose an obligation on countries to guarantee the health of their citizens. Rather, it guarantees the “highest attainable standard of health,” and requires every country to make all reasonable efforts, given their available resources, to increase their citizens’ access to the fundamental elements of a healthy life. Moreover, one of the critical components of the right to health is the principle of non-discrimination, recognizing how selected vulnerable groups, experience increased difficulty accessing these fundamental elements of a healthy life for a variety of reasons^8^. From a right to health perspective, the failure of a country to protect vulnerable groups from non-discrimination is unjustifiable [27].

The right to health was recognized in the 1946 Constitution of the WHO. It evolved significantly in recent years [27]. It has gained importance on the global scale particularly since the Cold War, when international attention turned towards poor health conditions in some countries, and most recently rose to prominence as a result of the HIV/AIDS pandemic [23]. Today, it is protected and recognized in a number of international treaties, the most important being the International Covenant on Economic, Social and Cultural Rights, which includes 166 countries parties [26].

Every country has ratified at least one human rights treaty recognizing the right to health [27]. As such, each is bound to make all reasonable efforts to protect it, given their available resources^9^. Failure to do so can, in principle, lead to political sanctions. In countries where access is an issue, a misunderstanding of the tenets of a human rights lens enhanced with specific cultural practices at stake in this paper may be conceived as violations of the right to health as unintended results. By so doing, there is a risk in delegitimizing overall values and traditions rooted for centuries [28].

### The value disconnect associated with the human rights discourse

Human rights are praised by many for setting a universal ideal of what the world “ought to be” like, thus providing a clear orientation for development frameworks and policies [24, 25]. However, this claimed universality has found itself at the center of increased controversy and debate. The dominance of Western countries on the political field, and consequently, in the shaping of the content of the international documents at the root of our current human rights framework, has led to skepticism in the ability for human rights discourse to best represent the perspectives of all countries [29].

The protection of women’s right to health is an area of human rights law, which is particularly dominated by Western perspectives [30]. Behaviour that is perceived as complying with the obligation to protect women’s right to health is generally construed narrowly. The way that the health effects of *machismo/marianismo* cultures and the practice of FGC/E are conceived as violations of international human rights law today [12] exemplifies this value disconnect between the dominant human rights discourse and perspectives of concerned countries. For instance, in 2011, the Special Rapporteur on the right of everyone to the enjoyment of the highest attainable standard of physical and mental health wrote that gender norms “entrenched in patriarchy,” similar to those resulting from *marianismo* and *machismo* value sets, perpetuated a violation of the right to health for women, and suggested that the best way of securing health rights for women in these communities would be to “erode” these norms ([31] p.63). Similarly, FGC/E has been widely condemned by much of the international community for years. It was re-conceptualized as a human rights violation at the 1993 World Conference on Human Rights in Vienna [32].

The potential harmful consequences these practices and values have on women’s access to healthcare and on their health in general are undeniable. However, the extent of the criticism these practices receive when being framed through a human rights lens merit further thoughts and discussion. Terms matter, especially when the definitions and associated meanings vary between and within communities [6, 9]. Nonetheless, the United Nations continues to define this operation – often viewed as a “social good” by practicing countries – as one of mutilation, illustrating a blatant disregard for many communities’ perspectives in favour of the Western interpretation of this act as one of condemnable violence.

The suggestion that *marianismo* culture is necessarily based on the perception of women as weak and submissive is not entirely accurate. While today’s manifestations of these values often perpetuate the marginalization of women, *marianismo* in some Latin American cultures initially stemmed from the religious belief of women as “semi-divine, morally superior, spiritually strong beings capable of sacrifice.” [33] p.3 Perhaps ironically, the submissiveness of women that emerged from this cultural belief initially highlighted female strength and superiority – an idea which today’s human rights discourse fails to address.

Similarly, *machismo* culture is conceived as promoting male dominance and violence over women by embodying the idea that a man must necessarily control his wife, both within the family and in sexual relationships, in order to be considered a man within society. The way *machismo* culture is currently practiced in many Latin American societies does promote a harmful gender dichotomy between men and women. However, what rights-based language fails to recognize is how *machismo* culture and the empowerment of women can co-exist. Instead, it promotes the abandonment of *machismo* culture altogether, without considering the nuances within machismo that could lead to a more equitable society for women.

*Machismo* culture lies on a spectrum. It is still practiced in many Latin American countries at the extreme, encouraging men to have multiple sexual partners and engage in unhealthy sexual practices in order to fit within the expected male gender role. Yet, an element of this culture, which is often overlooked, is the idea that men must be *gentlemen* – *caballeros* – and act as the protectors of their family [11]. Encouraging a shift from the prevalent cultural belief of the man as a dominant, sexual being towards one centered on protecting his family would likely be an effective means of promoting safer sexual practices – it finds a middle ground between preserving a long-standing culture while encouraging healthier behaviour. Instead, the human rights language insists on its abandonment altogether.

The Western influence on human rights discourse that has been at the center of its criticism undeniably rings true about cultural practices and values that adversely impact women’s health. Accordingly, the use of a rights-based language to address these traditions overlooks important nuances at the heart of their significance for the communities in which they are practiced, thus further contributing to the promotion of policies incompatible with the cultural environment that they are targeting. The ‘solutions’ proposed by human rights advocates – most of them centered on the abandonment of these practices and value systems - therefore become inefficient and harmful, perpetuating a de-legitimization of already marginalized cultures, fostering a growing distrust in human rights discourse and leading to the further victimization of girls and women.

### The consequences of human rights discourse: applying Mutua’s savage-victim-saviour construction to the right to health

International organizations and human rights advocacy groups have promoted strategies aimed at combatting practices, which act as barriers for women’s access to healthcare or adversely impact their health. The focus is various strategies to gradual abandonment, or “erosion,” of these practices. The prevention or criminalization of FGC/E, for instance, is at the heart of policies adopted to promote the right to health for women subject to this operation [19]. Cultural values viewed as promoting male dominance are equally condemned. As will be discussed further, the rationale for these policies is one rooted in good intentions - they aim to stop the violation of women’s rights and promote their well-being. However, such ‘solutions’ are minimally effective. For instance, legislation prohibiting FGC/E in certain countries and forbidding the medicalization of this practice in order to preserve women’s rights to dignity and autonomy of the person may be ignored or poorly enforced [28]. Similarly, programs aimed at promoting women’s right to access to healthcare through safe sex and condom use in countries with a heavy prevalence of HIV/AIDS are rarely effective, if they fail to address the socio-cultural and gender challenges underlying the disparity in health outcomes [1].

Makau Mutua brings forward a metaphorical “Savage-Victim-Saviour” (SVS) construction as an example of how the main actors in the human rights system – namely, the United Nations, Western countries, non-governmental organizations (NGO) and Western academics – promote an “unsettling” top-down approach to development [34]. He argues that such rights-based language harmfully pins the “good” of Western “saviour” intervention against the “evil” of “savage” cultural practices barring individuals from living a dignified life [34].

This SVS paradigm is applicable to the promotion of the right to health for women for the topic at hand. Using a women’s rights discourse to address women’s health in these communities unduly delegitimizes the cultural practices behind it, furthers the victimization of the women affected by it, and perpetuates a saviour-complex which celebrates the implementation of rights-based policies that, in reality, do not work as well as they should.

### The harm in girls and women’s rights: the de-legitimization of “savage” cultural practices and the further victimization of girls and women in the promotion of the right to health

According to the SVS construction, women’s rights language does not target the countries. Rather, it uses the countries as a proxy for attacking the “real savage,” that of cultural practices embedded within it, and which go against the Western conception of how a society should be [34]. It implies that practices which do not fit into a “good” narrative could be understood by default as “evil”. Women’s rights advocacy by international NGOs, rooted in Western influence and often governed by Western contributors, is such an example [29, 34]. Reports on women’s rights violations perpetuated by a state aimed at shaming countries before the international community, and stigmatize behaviour that – from the women’s rights lens – can be deemed unacceptable [34]. Framing FGC/E practices as “mutilation,” implying barbarism and deplorable violence, is an example of how women’s rights language may paint selected cultural practices as “savagery”. Yet, words do matter and the debate remains open and most relevant. In fact, the arguments are such that de-legitimizing selected cultural practices create a weariness moving women’s health and empowerment agenda further away from its initial goal of universal peace and international cooperation. These are some of the reasons that selected societies which view FGC/E practices as “social goods,” fundamental to the transmission of an age-old tradition within their culture, have become extremely critical of the human rights movement [6]. In turn, it becomes difficult for these communities to trust a narrative, which alienates their culture by defining it in, what they perceive as, a derogatory and offensive.

The WHO’s fact sheet on the promotion of the Right to Health cites the increased provision of condoms and the promotion of condom use [27]. It does not take into account the power dynamics within intimate relationships in the societies it seeks to help. Similarly, the most common international response to FGC/E practices and hinging on human rights norms is the promotion of legislation criminalizing it [18]. Indeed, these laws are often poorly enforced and monitored, often viewed as a form of coercion and suppression of values and traditions [28, 35]. Laws adopted against FGC/E practices in Senegal, for instance, were labeled as “symbolic,” with no intention to be enforced [36]. It is important to note that United Nations programs celebrate “milestone” anniversaries of the adoption of their recommendations against FGC/E. While this is important for awareness and education purposes, the effectiveness of the actual changes remain heavily debated [32].

The depiction of selected cultural practices as “savagery” is also associated with the notion of “victims” [34]. Women’s rights advocacy is, in part, based on the idea that there are victims of harmful practices in violation of their rights – it aims to prevent the creation of further victims, and to punish those who perpetuate this victimization. Ironically, the use of this language itself contributes to the very victimization it seeks to condemn. Women subject to *mariana* gender roles in selected countries in Latin America are framed as powerless. The image perpetrated by women exposed to FGC/E is similar - they are described as not being able to fully voice their opinions nor act on their personal preferences [32]. These statements are not without merit. The negative impacts on girls and women’s health should not be ignored. However, framing solutions through a lens that focuses on women’s weaknesses and, ‘woman as victim’, rather than playing to and working on their potential, is counterproductive. It inevitably furthers the victimization of women and perpetuates the idea that Western women’s rights ideals are the only solution to “saving” them from their damaging situation – that they cannot adequately contribute to their empowerment themselves. Efforts that begin to address the intersectionality of such social injustices are urgently needed.

## Community-based approaches to expanding women’s capabilities as a means of reconciling women’s rights with pervasive cultural norms

It is beginning to be widely agreed that a top-down approach will likely be moderately effective at best [37]. As do others, we we subscribe to solutions in which the gender gap in health conditions should come from within communities themselves [38]. Doing so would encourage the development of policies that reflect overall cultural values. We argue that an approach focusing on the expansion of women’s capabilities [13] – is a more effective means of improving women’s health than solely focused on human rights language. We posit that such an approach should focus on the creation of more opportunities for women, on how community-based efforts are likely to be most effective at transforming a society’s cultural context into one which can properly improve women’s health.

The capabilities approach to development was first advanced by Amartya Sen, and later elaborated by Martha Nussbaum, as a strong approach to development and gender justice. It focuses on promoting “what people are actually able to do and to be,” ([14] p.33) including the ability to achieve good bodily health and bodily integrity [39]. This approach frames the goal of development as allowing one to attain a position of equality - and places a strong emphasis on the importance each person’s ability to contribute to society. While the goals of a capabilities approach are similar to those of human rights language, the capabilities narrative is more focused on recognizing cultural pluralism by promoting opportunities and abilities [14].

In the context of women’s health, a capabilities approach has been defined as the promotion of women’s potential to achieve better health conditions [37]. Where culture acts as a barrier to the realization of this potential, we suggest that the best way of expanding women’s capabilities is not by abandoning the dominant cultural discourse entirely, but rather, in promoting its modification in a way which leaves room for women’s talents and resources to be made explicit and brought to the forefront. For instance, in *machismo* culture, shifting the dominant cultural role of the man to that of the *caballero,* responsible for protecting his family from harm, which might allow men to encourage women to use protection in sexual relationships [1]. Where men are expected to protect their families from harm, the potential for women to seek health care when they need it, or to negotiate safer sexual practices for their own security, increases – thus giving space for their capabilities to be of use while allowing the larger *machismo* values to nonetheless persist. Educating men about the potential harm of unsafe sexual practice while placing an emphasis on the role of the *caballero* as a protector, may have a positive impact on shifting the cultural discourse towards one which favours the protection of women. It has been recognized that a focus on male participation when challenging male hegemony within machismo communities is crucial [40].

Similarly, cultural values that underlie the practice of FGC/E are broad and include parents wanting the best health, marriage possibilities, and community respect for their daughters [41]. Community-based approaches to FGC/E that emphasize education about potential bodily consequences of FGC/E, especially in the context of recruitment of the community’s men and religious leaders, seem to have the most success as measured by women saying they would not circumcise their daughters [38].

In such a context, it is instructive to understand what community-based partnerships might mean. A case example is Tostan, a community-based initiative anchored in a human rights approach with community-based solutions to address the consequences of FGC/E [42]. Its success builds on the combination of human rights and community’s own values, including cultural arbiters, mediators, the fathers’ viewpoint and the clergy [38]. Similar case example is that of the complex role of foot-binding in China [43]. This suggests that the human rights language while persuasive on a world stage may not be so credible or relevant at a local level.

### Community-based approaches as channels for enhancing women’s health

While finding the benign in cultural values that from our Western perspective, harm women, is not an easy task, particularly when the values it seeks to address are strongly embedded and where they have existed in their current form for many years [44]. This shift in perspectives requires a sufficient understanding of the culture it addresses, and therefore, will be most effective when it comes from within the communities themselves. Approaches that consider knowledge, practices and capacities within the community, itself, will likely be the most effective at adapting the cultural norms, because they address “not what the community is doing, but why” ([44] p.231).

Providing women a platform to initiate change is an important element in improving their standard of living: “… a great deal about women’s health can be learned by letting women talk” ([30] p.30). The promotion of community-based education programs, which focus not on the practice itself, but on the corollary, harmful effects it has on women’s health, have met with some success in eliminating FGC/E without delegitimizing the cultural values behind the practice [45].

Community-Driven Development programs, which aim to empower local communities, include HIV/AIDS crisis management programs, while including stigma reduction strategies [46]. In Rwanda, for instance, increased funding in local NGOs allowed for culturally relevant and appropriate policies resulting in the adoption of innovative programs which invested in empowering women both economically and socially [47]. Political and social pushback towards initiatives which seek to challenge the *status quo* are inevitable. While selected cultural practices are pervasive, culture is not static [48]. Community-based approaches have shown promise, particularly those, which focus on education and raising awareness about the impacts of these practices with the community’s support, rather than condemning norms and practices themselves [9]. While shifting embedded cultural discourse may be a difficult and time-consuming process, its ability to preserve tradition while reaping benefits on women’s health remains a cause to consider.

## Conclusions

Promoting solutions to the gender gap in health conditions in selected regions of the world should undoubtedly remain on the international agenda, and the increased focus on this subject in recent years is reason to be optimistic about the future of gender equality in health [30]. However, the impact of prevailing cultural norms on this gender disparity in health conditions is often ignored, and the human rights narrative too often dismisses the importance of these cultural traditions. Cultures such as *marianismo* and *machismo* values in Latin American countries or those justifying the practice of female genital cutting/excision practices in selected countries in this world are deeply embedded in the societies they operate in, as well as in the identities of its women, men, girls and boys. Despite the potential harmful consequences on women’s health, recognizing the significance within their communities is important in order to foster a truly culturally plural world, and to promote the respect of the diverse societies that make up our international community today. The human rights discourse, linked to political power discourse, needs to engage in an endeavour of cultural understanding while being mindful of the pervasiveness risk of selected cultural practices. Culturally sensitive approaches to strengthen girls’ and women’s health need to reflect the spirit of community engagement and cultural pluralism that our global society should be committed to in a sustainable manner.

## Data Availability

Data sharing is not applicable to this article as no datasets were generated or analyzed during the current study.

## Abbreviations

FGC/E: Female genital cutting/excision
GNI: Gross national income
HIV/AIDS: Human immunodeficiency virus infection and acquired immune deficiency syndrome
NGO: Non-governmental organization
SVS: Savage-Victim-Saviour
USD: United States dollar
WHO: World Health Organization

## Footnotes

1 For the purposes of this paper, “low- and middle-income countries” are those considered to fall into these categories by the World Bank. Countries considered to be “low” income and “lower-middle income” are those with a gross national income (GNI) per capita under 3895 USD and those considered “upper-middle income” are those with a GNI per capita between 3896 USD and 12,055. See the World Bank, “New country classifications by income level: 2018-2019” (2018) online: https://blogs.worldbank.org/opendata/new-country-classifications-income-level-2018-2019.

2 The authors focus on the impacts of such cultural norms in the specific cases of HIV prevention in Latin America and in the perpetuation of FGC/E in Sub-Saharan Africa, but nonetheless recognize that such norms exist in a variety of forms in a number of different countries.

3 We note an important nuance to be considered with regards to FGC/E, in that the cultural values surrounding this practice vary between communities and may not always be motivated by discriminatory intentions. As noted by Einstein and Jacobs, 2018, some justifications for this practice include the belief that it will lead to positive health outcomes for the unborn fetus and can also come from a belief of female empowerment. FGC/E as a health inequity therefore refers particularly to the health outcome of this practice, in which women are subject to harmful health consequences due to this cultural practice.

4 Cianelli*, supra* note 2 at 298. It is important to note that, while the general principles of *machismo* and *marianismo* and their overall impacts on the social perceptions of women and men remain broadly applicable throughout most Latino cultures, the way this discourse specifically interacts within a given state varies from country to country. This paper focuses predominantly on its impacts in Peru, but nonetheless addresses the general principles of *machismo* and *marianismo* - applicable in other Latino nations - as well.

5 *Supra* note 1 at 748. It is important to note that these labels carry a very strong, derogatory connotation in Latin America. They are not terms that are used lightly.

6 The authors note that although this section addresses the continued practice of FGC/E in Sub-Saharan African countries because of its heavy prevalence in this area, still today, it is important to recognize that this practice continues to occur in a number of countries, including within Asia and in the Middle East, and is becoming increasingly pervasive across the globe due to increased migration.

7 The use of “rights talk” in nationalism and anti-colonialism movements in the history of a number of low-income countries is an example of the prominence of early human rights discourse. See Andrea Cornwall & Celestine Nyamu-Musembi, “Putting the ‘Rights-Based Approach’ to Development into Perspective” (2004) 25:8 Third World Quarterly 1415 at 1420.

8 For instance, women – especially in low-income regions – find themselves at the center of violence, poverty, and gender biases within society, as well as with very little power regarding their sexual activity. All of these factors can contribute to their differed experience with regards to access to health care than men (See *The Right to Health, supra* note 57 at 12).

9 See, for example, the *International Covenant on Economic, Social and Cultural Rights*, *supra* note 62 at art 2 (1). It is important to re-emphasize, however, that a State’s obligation to uphold the right to health is not an obligation to guarantee the health of its citizens (see *The Right to Health, supra* note 57 at 5).

## References

[1] Valencia-Garcia D, Starks H, Strick L, Simoni JM (2008). After the fall from grace: negotiation of new identities among HIV-positive women in Peru. Cult Health Sex.

[2] Amaro HJ, Raj A (2000). On the margin: power and Women’s HIV risk reduction strategies. Sex Roles.

[3] Cianelli R, Ferrer L, McElmurry BJ (2008). HIV prevention and low-income Chilean women: machismo, Marianismo and HIV misconceptions. Cult Health Sex..

[4] Thapan M (1997). Linkages between culture, education and Women's health in urban slums. Econ Polit Wkly.

[5] Ojanuga DN, Gilbert C (1992). Women’s access to health care in developing countries. Soc Sci Med.

[6] Vissandjée B, Denetto S, Migliardi P, Proctor J (2014). Female genital cutting (FGC) and the ethics of care: community engagement and cultural sensitivity at the interface of migration experiences. BMC Int Health Hum Rights.

[7] Cassman R (2008). Fighting to make the cut: female genital cutting studied within the context of cultural relativism. Nw J Int'l Hum Rts.

[8] United Nations (2015). Sustainable development goals 3: ensuring healthy lives and promote well-being for all at all ages.

[9] Vissandjée B, Kantiéb M, Aïthachimi L, Levine A. De l’Afrique au Canada: qu’en est-il des pratiques de l’excision et de l’infibulation? [Report]. Montreal: Centre de recherche et de formation, CLSC Côte-des-Neiges; 2009.

[10] Posner E. The case against human rights. The Guardian; 4 December 2014; Human Rights. Available from: https://www.theguardian.com/news/2014/dec/04/-sp-case-against-human-rights. [cited 2017 Sept 20].

[11] Kinzer S. End human rights imperialism now. The Guardian; 31 December 2010; Human Rights. Available from: https://www.theguardian.com/commentisfree/cifamerica/2010/dec/31/human-rights-imperialism-james-hoge [cited 2017 Oct 9].

[12] Lewis H (1995). Between Irua and ‘Female genital Mutilation’: feminist human rights discourse and the cultural divide. Harv Hum Rights J.

[13] Berik G, van der Meulen RY, Seguino S (2009). Feminist economics of inequality, development, and growth. Fem Econ.

[14] Nussbaum M (2003). Capabilities as fundamental entitlements: Sen and social justice. Fem Econ.

[15] Marian G, Kasznia-Brownb J, Paezc D, Mikhaild MN, Salamae DH, Bhatlaf N (2017). Improving women’s health in low-income and middle-income countries. Part I: challenges and priorities. Nucl Med Commun.

[16] Salyers BS (1998). Machismo/Marianismo attitudes, employment, education, and sexual behavior among women in Ecuador and the Dominican Republic. J Gender Cult Health.

[17] Moreno CL (2007). The relationship between culture, gender, structural factors, abuse, trauma, and HIV/AIDS for Latinas. Qual Health Res.

[18] World Health Organization. Female Genital Mutilation. [Place unknown]: WHO; 31 January 2018. Available from: https://www.who.int/en/news-room/fact-sheets/detail/female-genital-mutilation. [cited 2018 June 9].

[19] Costello S (2015). Female genital mutilation/cutting: risk management and strategies for social workers and health care professionals. Risk Manag Healthc Policy.

[20] Migliardi P, Vissandjée B, Einstein G (2011). At the crossroads: healthcare experiences of women with female genital cutting. Intersections.

[21] Abdulcadir J, Margairaz C, Boulvain M, Irion O (2011). Care of women with female genital mutilation/cutting. Swiss Med Wkly.

[22] Williams-Breault BD (2018). Eradicating female genital mutilation/cutting: human rights-based approaches of legislation, education, and community empowerment. Health Hum Rights.

[23] United Nations General Assembly (1948). Universal Declaration of Human Rights. Resolution 217 A (III).

[24] Cornwall A, Nyamu-Musembi C (2004). Putting the ‘rights-based approach’ to development into perspective. Third World Q.

[25] Ignatieff M (2001). The attack on human rights. Foreign Affairs.

[26] United Nations general assembly. International covenant on economic, social and cultural rights. Resolution 2200A (XXI). New York: UN; 16 December 1966. UN Treaty Series vol. 993. No. 14531, art. 12.

[27] World Health Organization (2008). The Right to Health. Fact Sheet No. 31.

[28] Cook RJ, Dickens BM, Fathalla MF (2011). Female Genital Cutting (Circumcision/Mutilation). Reproductive Health and Human Rights: Integrating Medicine, Ethics, and Law. Oxford Scholarship Online.

[29] Bukovská B (2008). Perpetrating good: unintended consequences of international human rights advocacy. Int J Human Rights.

[30] Inhorn MC (2006). Defining Women's health: a dozen messages from more than 150 ethnographies. Med Anthropol Q.

[31] Grover A (2011). Interim report of the Special Rapporteur on the right of everyone to the enjoyment of the highest attainable standard of physical and mental health, Human Rights Council.

[32] United Nations Children’s Fund (UNICEF) (2013). Female Genital Mutilation/Cutting: A statistical overview and exploration of the dynamics of change.

[33] Ehlers TB (1991). Debunking marianismo: economic vulnerability and survival strategies among Guatemalan wives. Ethnology..

[34] Mutua MW (2001). Savages, victims, and saviors: the metaphor of human rights. Harvard Intl LJ.

[35] Shell-Duncan B (2008). From health to human rights: female genital cutting and the politics of intervention. Am Anthropol.

[36] Shell-Duncan B, Hernlund Y, Wander K, Moreau A (2013). Legislating change? Responses to criminalizing female genital cutting in Senegal. Law Soc Rev.

[37] Ruger JP (2006). Toward a theory of a right to health: capability and incompletely theorized agreements. Yale J Law Humanit.

[38] Berg RC, Denison EM. A realist synthesis of controlled studies to determine the effectiveness of interventions to prevent genital cutting of girls. Paediatr Int Child Health. 2013;33(4):322–33. 10.1179/2046905513Y.0000000086 PMC3817579 24196703

[39] Nussbaum M (2000). Women’s capabilities and social justice. J Hum Dev.

[40] Sternberg P. Challenging machismo: promoting sexual and reproductive health with Nicaraguan men. Gend Dev. 2000;8(1):89–99. 10.1080/741923418 12349643

[41] Einstein G, Jacobson D, Lee J, Jordal M, Griffin G (2018). An analytic review of the literature on female genital circumcision/mutilation/cutting (FGC): the Möbius strip of body and society for women with FGC. Bodies, migration, (RE) constructive surgeries: making the gendered body in the globalized world.

[42] Tostan. Intersecting Issues. https://www.tostan.org/areas-of-impact/cross-cutting-gender-social-norms/. Accessed 20 May 2018.

[43] Mackie G (1996). Ending footbinding and infibulation: a convention account. Am Sociol Rev.

[44] Ensor J, Berger R, Adger WN, Lorenzoni I, O’Brien K (2009). Community-based adaptation and culture in theory and practice. Adapting to climate change - thresholds, values, governance.

[45] Toubia NF, Sharief EH (2003). Female genital mutilation: have we made progress?. Int J Gynaecol Obstet.

[46] The World Bank. Community-Driven Development. http://www.worldbank.org/en/topic/communitydrivendevelopment. Accessed 9 Dec 2017..

[47] Blankhart DM, Gorgens-Albino M, Mohammad N, Odutolu AO (2007). The Africa Multi-Country AIDS Program 2000–2006: Results of the World Bank’s Response to a Development Crisis.

[48] Narayan U (1977). Dislocating cultures: identities, traditions, and third world feminism.

